# Risky business: human-related data is lacking from Lyme disease risk models

**DOI:** 10.3389/fpubh.2023.1113024

**Published:** 2023-11-03

**Authors:** Erica Fellin, Mathieu Varin, Virginie Millien

**Affiliations:** ^1^Department of Biology, McGill University, Montréal, QC, Canada; ^2^Redpath Museum, McGill University, Montréal, QC, Canada; ^3^Centre d'Enseignement et de Recherche en Foresterie (CERFO), Québec City, QC, Canada

**Keywords:** blacklegged ticks, data synthesis, human-related, Lyme disease, risk assessment, risk map

## Abstract

Used as a communicative tool for risk management, risk maps provide a service to the public, conveying information that can raise risk awareness and encourage mitigation. Several studies have utilized risk maps to determine risks associated with the distribution of *Borrelia burgdorferi*, the causal agent of Lyme disease in North America and Europe, as this zoonotic disease can lead to severe symptoms. This literature review focused on the use of risk maps to model distributions of *B. burgdorferi* and its vector, the blacklegged tick (*Ixodes scapularis*), in North America to compare variables used to predict these spatial models. Data were compiled from the existing literature to determine which ecological, environmental, and anthropic (i.e., human focused) variables past research has considered influential to the risk level for Lyme disease. The frequency of these variables was examined and analyzed via a non-metric multidimensional scaling analysis to compare different map elements that may categorize the risk models performed. Environmental variables were found to be the most frequently used in risk spatial models, particularly temperature. It was found that there was a significantly dissimilar distribution of variables used within map elements across studies: Map Type, Map Distributions, and Map Scale. Within these map elements, few anthropic variables were considered, particularly in studies that modeled future risk, despite the objective of these models directly or indirectly focusing on public health intervention. Without including human-related factors considering these variables within risk map models, it is difficult to determine how reliable these risk maps truly are. Future researchers may be persuaded to improve disease risk models by taking this into consideration.

## Introduction

Tick borne diseases are caused by pathogens transmitted by infected ticks to an uninfected host. As the climate warms, it becomes possible for ticks to have increased abundance, survival, and feeding activity, and to expand their geographic distribution northwards (1, 2). Human activities and their impacts on natural habitats are further altering the distribution of these disease vectors, and human-wildlife contacts are increasing through means of socio-demographics (globalization, urbanization, etc...) and public health systems [vector control and other health interventions (3)]. Due to the constant changes in the environment, it is challenging to assess the risks associated with these infections without predictive modeling. Many current and future scenario predictive risk maps have been developed to monitor zoonotic infections for public health interventions (4–7). Epidemiologic risk maps are effective visualization tools used to identify geographical areas of high risk for disease transmission and potential future high-risk regions. Used as a communicative tool for risk management, these maps provide a service to the public, conveying information that can raise risk awareness and encourage mitigation strategies (8–10). Since climate and land use changes are constantly altering the dynamics between vector and host, continuous monitoring of the emergence and expansion of the disease vector is required. This is true of the spread of Lyme disease and other tick-borne diseases (2, 3).

In eastern North America, Lyme disease is typically caused by an infection of the spirochaete *Borrelia burgdorferi* via blacklegged ticks [*Ixodes scapularis* (11)]. In humans, the infection can result in a multisystem illness that substantially affects the individual's quality of life, if left untreated (12, 13). As a common and widespread disease (14–16) that can lead to severe conditions (17), effective communication tools for risk management of this infection are necessary.

Risk maps for Lyme disease in North America tend to focus on the impact climate change has on the distribution of blacklegged ticks (18–22), since these ectoparasites are vectors for *B. burgdorferi* (23) and their geographic range is increasing (1, 24). The geographic range of blacklegged ticks is heavily dependent on environmental variables such as temperature and precipitation (25–28). The geographical ranges of their hosts also play a major role (25). Blacklegged ticks require a single host for each life stage (29, 30), and will migrate with these hosts, such as small mammals, birds, and ruminants (31, 32).

Different variables have been considered to affect Lyme disease distribution. These include ecological variables related to blacklegged ticks [tick density, dispersion (33–35)] and their small mammal reservoir and migratory avian hosts (36, 37), as well as environmental variables such as temperature, humidity, and forest fragmentation (20, 38, 39). Recent risk map publications have adopted “One Health” approaches, which incorporate sociological, ecological, and biological knowledge into their research (40, 41). This approach aims to examine and integrate human-related or anthropic variables that may influence human health or risk (22, 42) including those variables beyond human demographics.

For instance, one may expect that human exposure and the risk of becoming infected by Lyme disease is also dependent on individual human behavior (e.g., knowledge, activity). As such, outdoor workers have been found to be more at risk for zoonotic diseases than those who are outdoors recreationally, due to their degree of exposure to the environment (43), and those with immune deficiencies may be more at risk for severe symptoms (44). Studies have included surveys of a population to gauge their knowledge on their risks to Lyme disease or tick infections (22, 42, 45, 46). Socio-economic status and ethnicity have also been found to play a role in Lyme disease risk (42, 47, 48). Knowing that not all individuals are equally at risk for being infected with diseases, including Lyme disease, it should be expected that studies in which Lyme disease risk maps are developed would include variables associated with human characteristics and behaviors. For these studies to be relevant to public health, variables associated with humans (i.e., social, economic, risk perception) should be taken into consideration, as these factors directly affect the risk posed to the public.

Here, we reviewed the literature to identify the variables past research has considered influential to the distribution of Lyme disease via blacklegged ticks in North America. Variables that researchers routinely included in risk models were examined, and those human variables that were often disregarded but may be informative were highlighted. By calling attention to the lack of human variables found in previous risk maps, future researchers may be persuaded to enhance models by including anthropic factors to improve disease risk prediction.

## Methods

### Collection of data

**Figure 1:**
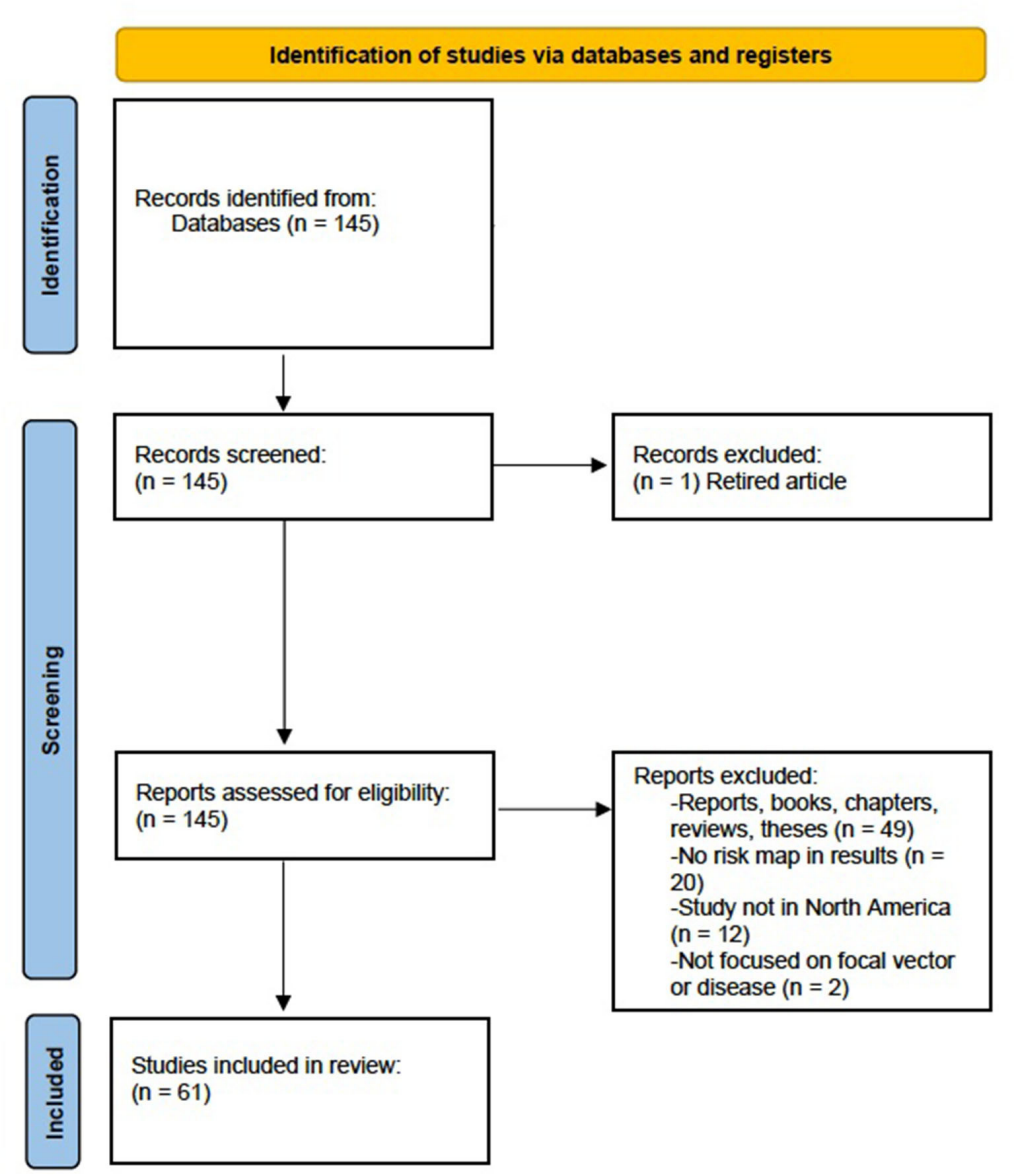
Preferred Reporting Items for Systematic Review and Meta-Analysis (PRISMA) flow diagram for systematic review which included searches of databases for risk maps related to Lyme disease and blacklegged tick (*Ixodes scapularis*) distributions.

We focused on past studies that are comparable due to similarities in geography [same continent, overlapping tick populations (49, 50)], disease vector, and spirochaete strain (*B. burgdorferi*). For this reason, we focused on one tick vector (black-legged tick) which is endemic to eastern North America (17). On March 31, 2023, a descriptive literature review was conducted following methods by Paré and Kitsiou (51) using Google Scholar, PubMed, and CrossRef with the inclusion criteria terms: “*Ixodes scapularis*,” “blacklegged ticks,” “risk map” (exact phrase), “Lyme disease,” “risk assessment,” and “*B. burgdorferi*.” This literature review concentrated on eastern North American (across Canada, the United States, and Mexico) risk assessments of Lyme disease transmitted by blacklegged ticks only. Geographical scale varied across studies, with some focusing on areas at the municipality, provincial/state, regional, or national scale. However, it should be noted that there are several risk assessments for Lyme disease concentrated in Europe and western North America where other tick species and spirochaete vectors can transmit Lyme disease (17, 20, 52). Only studies in which analyses included at least one risk map in their results was considered for this review, as we were specifically interested in comparing studies that produced risk map models to evaluate risk. We performed a systematic review following PRISMA (Preferred Reporting Items for Systematic Reviews and Meta-Analyses) guidelines (53). The initial search consisted of 145 studies published between 2000 and 2022 with the above criteria. Twenty studies were excluded, as they did not include a risk map in their results. Twelve more studies were excluded since their results were based outside of North America, another two studies were excluded as they focused on different vectors or bacteria and diseases and one article was removed from the literature review as it was retired and no longer considered relevant. Finally, 49 government reports, reviews, and theses were removed, leaving a total of 61 peer-reviewed articles meeting our criteria to be included in analyses (Figure 1; Supplementary Table 1).

Once the literature review was completed, data on the models within the articles were collected. This included collecting information on the number of ecological, environmental, and anthropic variables used in the studies to develop risk map models. Frequency counts for the number of each ecological, environmental, and anthropic variable in a risk map model were recorded. Ecological variables included variables that influence or dictate the relationship between an organism and its environment (i.e., tick occurrence, *B. burgdorferi* prevalence). Environmental variables included natural resource factors that define an ecosystem or habitat (i.e., temperature, humidity, land cover). Anthropic variables were defined as variables related to human beings (i.e., population density, sex, age). As Lyme disease is primarily transmitted via tick vectors (11), these observations do not provide any information on how the disease circulates within a human population. Further, certain predictor variables were simplified to allow comparisons more easily. For example, forest cover and vegetation index were categorized together, as were vapor pressure and humidity, elevation and altitude, human population size and density, and tick abundance and density (Supplementary Table 2).

Additional elements that characterized these risk models were collected and recorded, including the year of publication (Supplementary Table 3), the study's focal location (Country), Map Type (predictive vs. surveillance), the period of the study (year), the focal Scale of study (local, regional, national), the distribution of the study considered– vector (tick) vs. host (human or otherwise) vs. vector and host (both considered)—the Tick Surveillance methodology used for the model (passive vs. active vs. no tick surveillance), and the Tick Life Stage the tick data was based on (immature ticks vs. adult vs. all stages vs. no tick data; Table 1; Supplementary Table 4). Map Type (predictive vs. surveillance) was also included, whereby “predictive” maps referred to future predictive map models, as they predict future scenarios, while current predictive maps will be referred to as “surveillance” models, as they pertain to current risks.

**Table 1:** The cumulative number of anthropic, environmental, and ecological variables used across map elements in the reviewed studies.

**Map element**		**Anthropic**	**Environmental**	**Ecological**
Map Type	Surveillance (38)	17	40	52
	Predictive (25)	3	62	30
Map distribution	Host (16)	14	24	19
	Vector (33)	5	62	38
	Host and vector (15)	2	16	26
Map scale	Local (38)	14	58	54
	Regional (17)	6	30	18
	National (9)	0	14	10
Tick life stage	Adult (3)	1	3	4
	Immature (12)	1	30	16
	All stages (32)	10	51	49
	No tick data (16)	8	18	13
Tick surveillance	Passive (18)	4	23	23
	Active (19)	5	27	26
	Active and passive (10)	0	15	16
	No surveillance data (18)	14	22	10
Country	Canada (27)	12	41	34
	USA (31)	5	55	45
	USA and Canada (3)	2	4	1
	USA and Mexico (1)	1	0	1
	USA, Canada, and Mexico (1)	0	2	1

The total number of variables for each variable group is the sum of those variables used across studies (n = 61). Numbers in brackets indicate the number of studies for that specific map element.

USA, United States of America.

Most risk map models are predictive as they use modeling to create these maps. However, some models are used to predict risks associated with potential future geographical ranges and distributions of Lyme disease (18, 38, 39, 54, 55), while others are used to predict the current risk or prevalence (56–59). In addition, Map Scale was considered and categorized by whether the study focused their spatial scale by country (national), province or state (regional), or a smaller unit (e.g., census division; local).

### Synthesis of data

A non-metric multidimensional scaling (NMDS) analysis was performed in R [(60); version 1.4.1717] using the “*vegan*” package (version 2.6-2) to determine any significant differences in the frequency of ecological, environmental, and anthropic variables used across studies (*n* = 61), depending on their map elements: Map Type, Map Scale, Map Distribution, Year, Tick Life Stage, Map Surveillance method, and Country. Here, the NMDS, which is commonly used as an ordination for community ecology (61), was performed where “sites” were the individual studies, and “environmental data” were the map elements that influenced the abundance of “species” (ecological, environmental, and anthropic variables).

Linear models were then used to determine if groups of variables (ecological, environmental, and anthropic) differed in frequency across the different map elements identified by the NMDS. A binomial linear regression was conducted with Map Type as the response variable (surveillance vs. predictive) and the number of ecological, environmental, and anthropic variables used in the studies as predictors. An ordinal linear regression with the “logit” function and “equidistant” threshold was conducted (62) with Map Distribution (as a factor) as the response variable (host vs. vector. vs. host and vector), using the package “*ordinal*” [version 2019.10 (63)]. *Post-hoc* tests were conducted, and box plots were used to visualize variation within those map elements that were identified as significant by the NMDS.

## Results

**Figure 2:**
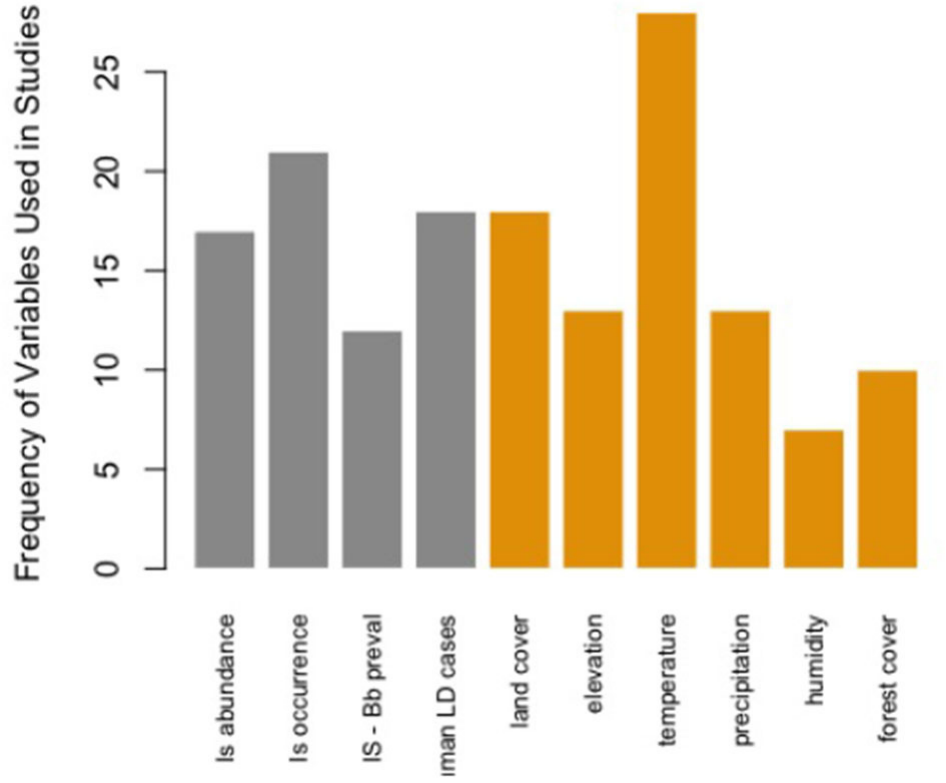
Frequency of the top 10 variables used within the 61 Lyme disease risk map models in North American studies published between 2000 and 2022. Is abundance, *Ixodes scapularis* abundance; Is occurrence, *Ixodes scapularis* presence/absence; Is—Bb preval = *Borrelia burgdorferi* prevalence in *Ixodes scapularis*; LD, Lyme disease.

**Figure 3:**
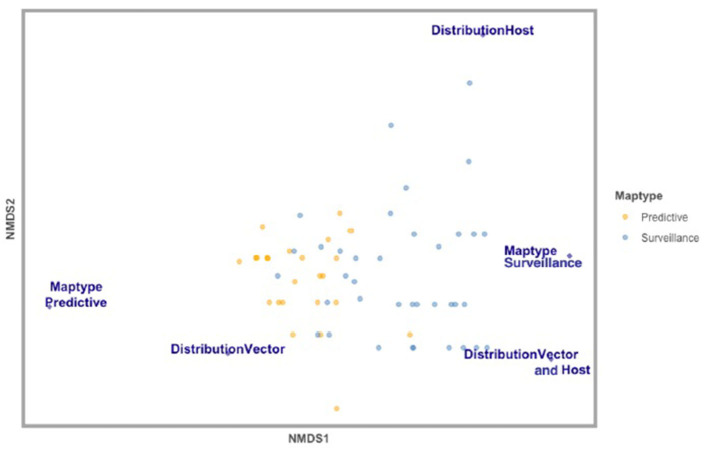
Non-metric multidimensional scaling (NMDS) analysis of map elements classifying studies based on the number of ecological, environmental, and anthropic variables incorporated in each study included in this review (*n* = 61). Variables displayed are significantly distinct variables across studies—Map Type (surveillance vs. predictive), Map Distribution (vector vs. host vs. vector and host), and Map Scale (local, regional, national). Studies that developed Predictive risk maps are in yellow, and studies that developed Surveillance risk maps are in blue.

Comparatively, certain map elements were more frequently used across the studies assessed in this literature review. For Map Type, there were more predictive maps than surveillance. For Map Distribution, there were more vector (blacklegged tick) distributions considered than human or both human and tick distributions. Maps at the local scale were most common across studies. All stages for Tick Life Stage were most frequently considered vs. specific life stages. Both active and passive data for Tick Surveillance was most frequently included in risk map models and most studies in this assessment were based in the United States (Table 1). The most common variable included in Lyme disease or blacklegged tick risk maps was temperature (*n* = 28 studies). Of the 10 most frequently used variables in these map models, six were environmental variables, four were ecological, and none were anthropic (Figure 2). The results of the NMDS analysis suggested that there were differences in the frequency of ecological, environmental, and anthropic variables used across Map Type and Map Distribution (Table 2; Figure 3; Supplementary Table 5).

**Table 2:** Non-metric multidimensional scaling (NMDS) goodness of fit results of map elements classifying studies based on the frequency of ecological, environmental, and anthropic variables incorporated in each risk map model (*n* = 61).

**Variables**	***r* ^2^**	**Pr (>*r*)**
Year of publication	0.0421	0.289
Map Type	0.2268	**0.001**
Distribution	0.1221	**0.012**
Scale	0.0350	0.372
Tick life stage	0.0415	0.811
Tick surveillance	0.0352	0.653
Country	0.0468	0.730

Variables include Year of publication, Map Type, Map Distribution, Tick Life Stage, Country. Number of permutations = 999. Statistically significant results are in bold.

**Figure 4:**
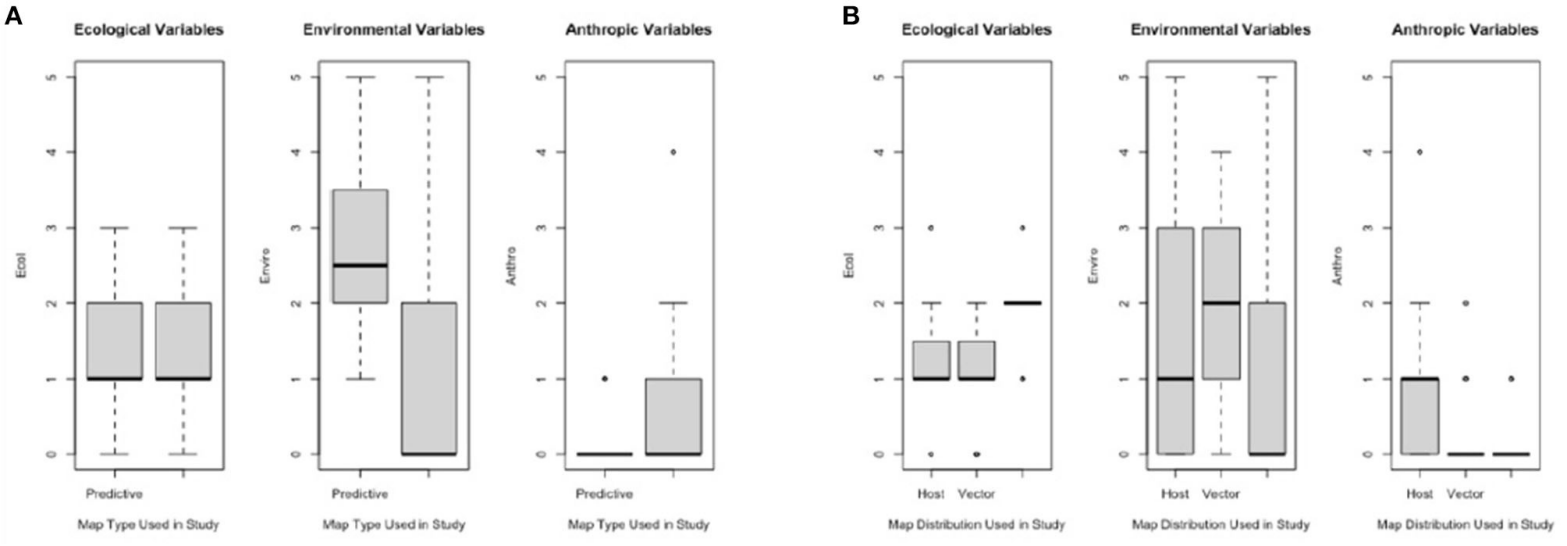
Frequency of ecological, environmental, and anthropic variables used across studies (*n* = 61) for varying map elements: **(A)** Map Types—predictive (future scenario; *n* = 24) and surveillance (current distribution; *n* = 37), and **(B)** Map Distributions—host (*n* = 15), vector (*n* = 15), vector (*n* = 32), or both vector and host (V & H; *n* = 14).

When comparing Map Types (surveillance vs. predictive), the difference in use of environmental variables was statistically significant (*p* < 0.0173), with predictive maps using more of these types of variables. For both types of risk maps, anthropic variables were rarely used (Table 3; Figure 4A). Comparing across Map Distributions, studies that included host distributions in risk models considered more anthropic variables than studies that included vector distributions (*p* < 0.0152). Meanwhile studies that included both host and vector distributions tended to include more ecological variables (*p* < 0.0291; Table 4; Figure 4B). There was no significant difference across Year of publication, Surveillance Type used, Map Scale, Country of study origin, or Tick Life Stage focused on in studies (Table 2).

**Table 3:** General linear model results for Map Type ~ anthropic + environmental + ecological, where family = “binomial” and the independent variables are counts (the number of variables in each category).

**Variable type**	**Estimate**	**Standard error**	***z* value**	**Pr (>|*z*|)**
(Intercept)	0.5878	0.8051	0.730	0.4653
Anthropic	0.7311	0.6579	1.111	0.2665
Environmental	−0.6633	0.2129	−3.115	**0.0018**
Ecological	0.6571	0.5070	1.296	0.1950

Null deviance = 81.772 on 60 degrees of freedom, residual deviance = 64.676 on 57 degrees of freedom. AIC: 72.676, n = 61. Statistically significant results are in bold.

**Table 4:** Cumulative link model results for map distribution ~ anthropic + environmental + ecological, where link = “logit,” threshold = “equidistant” and the independent variables are counts (the number of variables in each category).

**Variable type**	**Estimate**	**Standard error**	***z* value**	**Pr (>|*z*|)**
Anthropic	−1.7432	0.6016	−2.897	**0.00376**
Environmental	−0.3410	0.1790	−1.905	0.05681
Ecological	1.0577	0.4504	2.348	**0.01887**
Threshold-coefficients				
Threshold	−1.1201	0.7432	−1.507	–
Spacing	2.9951	0.4817	5.657	–

logLik = −51.82, AIC = 113.64, n = 61. Statistically significant results are in bold.

## Discussion

Although there is a plethora of literature dedicated to identifying factors that can influence an individual's risk for Lyme disease via tick vectors directly and indirectly (48, 64, 65), risk maps that demonstrate the spatial breadth of these risks are less common. The results of this review have shown that within this subset of risk maps, there is no standardized risk score, or variable being used across studies. Temperature was the most common variable used in risk map models, however, it was included in less than half of the risk maps considered. There is extensive research on the relationship between blacklegged ticks and temperature, as it affects a tick's development, survival, and host-seeking behavior (19, 66, 67). These are significant factors that influence tick abundance and distribution, and therefore influence the distribution and incidence of Lyme disease (19, 68). In general, the most common variables included in Lyme disease (or blacklegged tick) risk map models were environmental and ecological, while anthropic were lacking. It should be noted that certain studies, such as Slatculescu et al. (69) considered many anthropic variables, including population density, walkability scores in an urban setting, median income, and drew conclusions about the contribution of an individual's variability on their Lyme disease risk, however, they did not express these results spatially in a risk map model. Similarly, several other ecological variables often studied and considered influential to Lyme disease and/or blacklegged tick distributions were rarely included in these risk map models. For instance, blacklegged tick distributions are affected by reservoir and migratory host distributions (36, 70) while Lyme disease distributions can be influenced by the genetic diversity of *B. burgdorferi* strains (49, 71). Without considering these variables within risk map models, it is difficult to determine how reliable these risk maps truly are.

Depending on the Map Type and Map Distribution used, studies significantly differed in the number of ecological, environmental, and anthropic variables used to produce risk maps. Studies that utilized different Map Types also significantly varied in their usage of environmental variables, where studies that produced predictive maps used this group of variables more often. Interestingly, predictive map studies also used anthropic variables less often. This suggests that when research focuses on future scenarios, they are reliant on how the environment may change, but do not consider human behavior. Although it is difficult to assess future trends in human behavior, it is still possible to include anthropic variables such as socio-economic status and demographic information (22) to gain a better understanding of the patterns in human attributes that could influence their risk for Lyme disease.

Across Map Distribution types, studies that included host distributions in their risk maps had more anthropic variables than other distributions (vector only or host and vector distributions). Risk maps that included vector only or both host and vector distributions tended to include more ecological variables. As most host distributions in these risk map models were human distributions, it is logical for human attributed variables to be included in these models. Meanwhile, research that incorporated both host and vector distributions, or only vector distributions may be more likely to include variables from the environment, as they are less directly focused on Lyme disease risk for humans specifically. Rather, they use the result of their risk map model that included disease vector distributions to indirectly make conclusions for public health risks (18, 19, 42).

This review was limited to research that included risk maps as results within a study, excluding studies that included maps within introductions and methods for context. In some cases, a single variable (e.g., tick distribution) was used as a proxy for Lyme disease distribution and correlated with environmental or human-related data [e.g., forest cover, urban development; (36, 72)]. Other studies considered several variables, but these distribution models were not applied to develop risk maps (38, 73). It should also be noted that despite only 20 incidences of human-related variables being included in these studies' models overall (Table 5), more recent research has begun to consider general public influence within their studies by including citizen science or “Google trends” to determine blacklegged tick and Lyme disease distributions (37, 89, 90). Other human demographic data can be acquired through public government agencies such as population densities and household incomes (69, 74–76). More personal and individualistic information can be acquired via questionnaire surveys (22, 42, 45, 46).

**Table 5:** List of peer reviewed articles included in the literature review that incorporated anthropic variables in their risk assessments where a map was produced as a result.

**Paper ID**	**Anthropic variables used**	**References**
MP9	- Human population density	Lieske and Lloyd (74)
MP15	- Human population size per county	Bisanzio et al. (76)
MP18	- Household income	Little et al. (75)
MS3	- Age and sex of Lyme disease patients	Tutt-Guerette et al. (84)
MS8	- Human population density - Hiking behavior and Lyme awareness survey	Tadiri et al. (45)
MS12	- Human population size per census division	Gasmi et al. (85)
MS17	- “Lifestyle” categories based on surveys	Ozdenerol et al. (42)
MS18	- Sociobehaviours; preventive behavior score, knowledge score, and risk perception score - Human population density	Bouchard et al. (22)
MS21	- Behavioral risk factors - Reported tick exposure (by survey respondents)	Aenishaenslin et al. (46)
MS25	- Human population density	Self et al. (86)
MS26	- Human population density	Glavanakov et al. (87)
MS28	- Human population density - Google trends for Lyme disease focused key words	Kutera et al. (88)
MS35	- Human population density	Diuk-Wasser et al. (36)
MS36	- Human population density	Larsen et al. (72)

Specific anthropic variables are described below along with their source.

There is a clear understanding that environmental factors heavily influence the distribution of diseases and their vectors (21, 52, 77, 78). However, very few studies consider that human related factors may also influence these distributions (22, 69). Further, human population growth, urbanization, and travel can affect vector-borne distributions (3), particularly when human movement or land development influences animal movement, effectively altering the host dynamics of tick vectors (79, 80). This is concerning, as the risks related to Lyme disease directly affect human health, and human behavior can affect infection rates (46, 69, 81, 82). Predictive Map Type risk maps especially ignore how human-related factors may influence Lyme disease risks for humans, and this may be due to studies focusing on *B. burgdorferi* distribution rather than infection rates.

Overall, it was found that risk maps that focused on Lyme disease and blacklegged tick distributions differed across the types of variables used according to the study goal and intended use of the map. Because of these inconsistencies, it is difficult to compare and validate these models to accurately forecast Lyme disease risks geographically or temporally. At the same time, geospatial data related to anthropic factors can be difficult to acquire. Our results bring attention to the fact that there is no consistent “risk” variable or assessment across studies, likely because these studies tend to vary in specific objectives, despite the general intent of public health intervention. For this reason, risk maps should be scrutinized more thoroughly. As our knowledge on blacklegged ticks and *B. burgdorferi* increases, we must continually re-assess how risk models have predicted their geographic distributions over time. Differences in tick exposure patterns and Lyme disease risk is likely across regions and can depend on the scale and socioeconomic factors included in the assessment (69). Future studies should consider improvements for forecasting these risks, as well as exploring risk assessments beyond comparison of blacklegged ticks and Lyme disease. Expanding the scope to other tick-borne diseases or co-infections of other bacteria including *Babesia* and *Anaplasma* sp. (16, 83) may demonstrate further patterns with spatial risk models.

## Data availability statement

The original contributions presented in the study are included in the article/Supplementary material, further inquiries can be directed to the corresponding author.

## Author contributions

EF: conceptualization, methodology, formal analysis, investigation, data curation, writing—original draft, visualization, and project administration. MV: resources, writing—review and editing, project administration, and funding acquisition. VM: conceptualization, resources, writing—review and editing, supervision, project administration, and funding acquisition. All authors contributed to the article and approved the submitted version.
