# Supplementary material for: Risky business: human-related data is lacking from Lyme disease risk models
Source: Front Public Health. 2023 Nov 3;11:1113024. doi: 10.3389/fpubh.2023.1113024 (PMC10662633; doi:10.3389/fpubh.2023.1113024)
Supplement: Supplementary file 1 [file Table_1.docx]

**Supplementary Tables**

**Supplementary Table 1.** Research paper identifications and their respective article citations.

| Paper ID | Citation |
| --- | --- |
| MP1 | Baldwin, H, Landesman, WJ, Borgmann-Winter, B, Allen, D. (2022). A geographic information System Approach to Map Tick Exposure Risk at a Scale for Public Health Intervention. J. Med. Entomol. 59(1): 162–172. doi: 10.1093/jme/tjab169 |
| MP2 | Ogden, NH, St-Onge, L, Barker, IK, Brazeau, S, Bigras-Poulin, M, Charron, DF, Francis, CM, Heagy, A, Lindsay, LR, Maarouf, A, Michel, P, Milord, F, O'Callaghan, CJ, Trudel, L, Thompson, RA. (2008). Risk maps for range expansion of the Lyme disease vector, Ixodes scapularis, in Canada now and with climate change. Int. J. Health Geogr. 7(24): 1-5. doi:10.1186/1476-072X-7-24 |
| MP3 | Leighton, PA; Koffl, JK; Pelcat, Y; Lindsay, LR; Ogden, NH. (2012). Predicting the speed of tick invasion: An empirical model of range expansion for the Lyme disease vector Ixodes scapularis in Canada. J. App. Ecol. 49(2): 457-464 doi: 10.1111/j.1365-2664.2012.02112.x |
| MP4 | Ogden, NH, Maarouf, A, Barker, IK, Bigras-Poulin, M, Lindsay, LR, Morshed, MG, O'Callaghan, CJ, Ramay, F, Waltner-Toews, S, Charron, DF. (2006). Climate change and the potential for range expansion of the Lyme disease vector Ixodes scapularis in Canada. Int. J. Parasitol. 36: 63-70. doi:10.1016/j.ijpara.2005.08.016 |
| MP5 | Guerra, M, Walker, E, Jones, C, Paskewitz, S, Cortinas, MR, Stancil, A, Beck, L, Bobo, M, Kitron, U. (2002). Predicting the Risk of Lyme Disease: Habitat Suitability for Ixodes scapularis in the North Central United States. Emerg. Infect. Dis. 8(3): 289-297. doi: 10.3201/eid0803.010166 |
| MP6 | Slatculescu, AM, Clow, KM, McKay, R, Talbot, B, Logan, JL, Thickstun, CR, Jardine, CM, Ogden,NH, Knudby, AJ, Kulkarni, MA. (2020). Species distribution models for the eastern black legged tick, Ixodes scapularis, and the Lyme disease pathogen, Borrelia burgdorferi, in Ontario, Canada. PLoS ONE. 15(9): 30238126. doi: 10.1371/journal.pone.0238126 |
| MP7 | Gabriele-Rivet, V, Koffi, JK, Pelcat, Y, Arsenault, J, Cheng, A, Lindsay, LR, Lysyk, TJ, Rochon, K, Ogden, NH. (2017). A risk model for the Lyme disease vector Ixodes scapularis (Acari: Ixodidae) in the Prairie Provinces of Canada. J. Med. Entomol. 54(4): 862-868. doi: 10.1093/jme/tjx036 |
| MP8 | Zhang, L, Ma, D, Li, C, Zhou, R, Wng, J, Liu, Q. (2022). Projecting the potential distribution areas of Ixodes scapularis (Acari: Ixodidae) driven by climate change. Biol. 11:107. doi: 10.3390/biology11010107 |
| MP9 | Lieske, DJ, Lloyd, VK. (2018). Combining public participatory surveillance and occupany modelling to predict the distributional response of Ixodes scapularisto climate change. Ticks. Tick. Borne. Dis. 9: 695-706. doi: 10.1016/j.ttbdis.2018.01.018 |
| MP10 | Diuk-Wasser, MA, Hoen, AG, Cislo, P, Brinkerhoff, R, Hamer, SA, Rowland, M, Cortinas, R, Vourc'h, G, Melton, F, Hickling, GJ, Tsao, JI, Bunikis, J, Barbour, AG, Kitron, U, Piesman, J, Fish, D. (2012). Human risk of infection with Borrelia burgdorferi, the Lyme disease agent, in Eastern United States. Am. J. Trop. Med. Hyg. 86(2): 320-327. doi:10.4269/ajtmh.2012.11-0395 |
| MP11 | Dumic, I, Severnini, E. (2018). "Ticking bomb": The impact of climate change on the incidence of Lyme disease. Can. J. Infect. Dis. Med. Micro. 2018: 1-10. doi: 10.1155/2018/5719081 |
| MP12 | Brownstein, JS, Holford, TR, Fish, D. (2003). A climate-based model predicts the spatial distribution of the Lyme disease vector Ixodes scapularis in the United States. Env. Health Persp. 111(9): 1152-1157. doi: 10.1289/ehp.6052 |
| MP13 | Wu, Z, Duvvuri, VR, Lou, Y, Ogden, NH, Pelcat, Y, Wu, J. (2013). Developing a temperature-driven map of the basic reproductive number of the merging tick vector of Lyme disease Ixodes scapularis in Canada. J. Theoretical Bio. 319: 50-61. doi: 10.1016/j.jtbi.2012.11.014 |
| MP14 | Soucy, JR, Slatculescu, AM, Nyiraneza, C, Ogden, NH, Leighton, PA, Kerr, JT, Kulkarni, MA. (2018). High-resolution ecological niche modeling of Ixodes scapularis ticks based on passive surveillance data at the northern frontier of Lyme disease emergence in North America. Vector. Borne. Zoo. Dis. doi: 10.1089/vbz.2017.2234 |
| MP15 | Bisanzio, D, Fernandez, MP, Martello, E, Reithinger, R, Diuk-Wasser, MA. (2020). Current and Future Spatiotemporal Patterns of Lyme Disease Reporting in the Northeastern United States. JAMA Network Open. 3(3): e200319. doi: 10.1001/jamanetworkopen.2020.0319 |
| MP16 | Brownstein, JS, Holford, TR, Fish, D. (2005). Effect of climate change on Lyme disease risk in North America. EcoHealth. 2: 38-46. doi: 10.1007/s10393-004-0139-x |
| MP17 | Ripoche, M, Bouchard, C, Irace-Cima, Leighton, PA, Thivierge, K. (2022). Current and future distribution of Ixodes scapularis ticks in Quebec: Field validation of a predictive model. PLoS ONE. 17(2): e0263243. doi: 10.1371/journal.pone.0263243 |
| MP18 | Little, EAH, Anderson, JF, StaXord III, KC, Eisen, L, Eisen, RJ, Molaei, G. (2019). Predicting spatiotemporal patterns of Lyme disease incidence from passively collected surveillance data for Borrelia burgdorferi sensu lato-infected Ixodes scapularis ticks. Ticks. Tick. Borne. Dis. 10(5): 970-980. doi:10.1016/j.ttbdis.2019.04.010 |
| MP19 | Diuk-Wasser, Ma, Vourc'h, G, Cislo, P, Hoen, AG, Melton, F, Hamer, SA, Rowland, M, Cortinas, R, Hickling, GJ, Tsao, JI, Barbour, AG, Kitron, U, Piesman, J, Fish, D. (2010). Field and climate-based model for predicting the density of host-seeking nymphal Ixodes scapularis, an important vector of tick-borne disease agents in the eastern United States. Global Ecol. Biogeogr. 19: 504-514. doi: 10.1111/j.1466-8238.2010.00526.x |
| MP20 | Johnson, TL, Boegler, KA, Clark, RJ, Delorey, MJ, Bjork, JKH, Dorr, FM, Schiffman, EK, Neitzel, DF, Monaghan, AJ, Eisen, RJ. (2018). An acarological risk model predicting the density and distribution of host-seeking Ixodes scapularis nymphs in Minnesota. Am. J. Med. Hyg. 98(6): 1671-1682. doi:10.4269/ajtmh.17-0539 |
| MP21 | McPherson, M, Garcia-Garcia, A, Cuesta-Valero, FJ, Beltrami, H, Hansen-Ketchum, P, MacDougall, D, Ogden, NH. (2017). Expansion of the Lyme disease vector Ixodes scapularis in Canada inferred from CMIP5 climate projections. Enviro. Health. Persp. 125(5): e057008. doi: 10.1289/EHP57 |
| MP22 | Couper, LI, MacDonald, AJ, Mordecai, EA. (2020). Impact of prior and projected climate change on US Lyme disease incidence. Glob. Health. Biol. 27(4): 738-754. doi: 10.1111/gcb.15435 |
| MP23 | Ogden, NH, Radojevic, M, Wu, X, Duvvuri, VR, Leighton, PA, Wu, J. (2014). Estimated effects of projected climate change on the basic reproductive number of the Lyme disease vector Ixodes scapularis. Environ. Health Perspect. 122: 631–638. doi: 10.1289/ehp.1307799 |
| MP24 | Feria-Arroyo, TP, Castro-Arellano, I, Gordillo-Perez, G, Cavazos, AL, Vargas-Sandoval, M, Grover, A, Torres, J, Medina, RF, Pérez de León, AA, Esteve-Gassent, MD. (2014). Implications of climate change on the distribution of the tick vector Ixodes scapularis and risk for Lyme disease in the Texs-Mexico transboundary region. 7:199. |
| MS1 | Johnson, TL, Bjork, JKH, Neitzel, DF, Dorr, FM, SChiffman, EK, Eisen, RJ. (2016). Habitat suitability model for the distribution of Ixodes scapularis (Acari: Ixodidae) in Minnesota. J. Med. Entomol. 53(3): 598-606. doi: 10.1093/jme/tjw008 |
| MS2 | Frank, C, Fix, AD, Pena, CA, Strickland, GT. (2002). Mapping Lyme Disease Incidence for Diagnostic and Preventative Decisions, Maryland. Emerg. Infect. Dis. 8(4): 427–429. doi: 10.3201/eid0804.000413 |
| MS3 | Tutt-Guerette, M, Yuan, M, Szaroz, D, McKinnon, B, Kestens, Y, Guillot, C, Leighton, P, Zinszer, K. (2021). Modelling spatiotemporal patterns of Lyme disease emergence in Quebec. Int. J. Environ. Res. Public Health. 18: 9669. doi: 10.3390/ijerph18189669 |
| MS4 | Clow, KM, Ogden, NH, Lindsay, LR, Michel, P, Pearl, DL, Jardine, CM. (2016). Distribution of ticks and the risk of Lyme disease and other tick-borne pathogens of public health significance in Ontario, Canada. Vector Borne Zoonotic Dis. 16(4): 215-22. doi: 10.1089/vbz.2015.1890 |
| MS5 | Egizi, A, Roegner, V, Faraji, A, Healy, SP, Schulze, TL, Jordan, RA. (2018). A historical snapshot of Ixodes scapularis-borne pathogens in New Jersey ticks reflects a changing disease landscape. Tick. Tick. Borne. Dis. 9: 418-426. doi: 10.1016/j.ttbdis.2017.12.009 |
| MS6 | Kotchi, SO, Bouchard, C, Brazeau, S, Ogden, NH. (2021). Earth observation-informed risk maps of the Lyme disease vector Ixodes scapularis in central and eastern Canada. Remote Sens. 13: 524. doi: 10.3390/rs13030524 |
| MS7 | Pasternak, AR, Palli, SR. (2022). Mapping distributions of the Lyme disease vector, Ixodes scapularis, and spirochete, Borrelia burgdorferi, in Kentucky using passive and active surveillance. Ticks. Tick. Borne. Dis. 13(): 101885. |
| MS8 | Tadiri C, Ainsworth N, De Bono N, Gavin S, Li J,Milbers K, Sardinas L, Schwartz N. 2011 Assessmentof human health risk for Lyme disease in a eriurban park in southern Quebec. McGill Sci. Undergrad. Res. J. 6: 56–61. |
| MS9 | c, B, Mark-Carew, M. (2017). Using exploratory data analysis to identify and predict patterns of human Lyme disease case clustering within a multistate region, 2010-2014. Spatial. Spatio-temp. Epidemiol. 20: 35-43. doi: 10.1016/j.sste.2016.12.003 |
| MS10 | Atkinson, SF, Sarkar, S, Avina, A, Schuermann, JA, Williamson, P. A determination of the spatial concordance between Lyme disease incidence and habitat probability of its primary vector Ixodes scapularis (black-legged tick). Geo. Health. 9(1): 203-212. |
| MS11 | Estrada-Pena, Agustin. (2009). Diluting the dilution effect: a spatial Lyme model provides evidence for the importance of habitat fragmentation with regard to the risk of infection. Geospatial Health. 3(2): 143-155. doi: 10.4081/gh.2009.217 |
| MS12 | Gasmi, S, Ogden, NH, Ripoche, M, Leighton, PA, Lindsay, RL, Nelder, MP, Rees, E, Bouchard, C, Vrbova, L, Rusk, R, Russell, C, Pelcat, Y, Mechai, S, Kotchi, S, Koffi, JK. (2019). Detection of municipalities at-risk of Lyme disease using passive surveillance of Ixodes scapularis as an early signal: A provincespecific indicator in Canada. PLoS ONE. 14(2): e0212637. doi: 10.1371/journal.pone.0212637 |
| MS13 | Chilton, NB, Curry, PS, Lindsay, LR, Rochon, K, Lysyk, TJ, Dergousoff, SJ. (2020). Passive and active surveillance for Ixodes scapularis (Acari: Ixodidae) in Saskatchewan, Canada. J. Med. Entomol. 57(1): 156-163. doi: 10.1093/jme/tjz155 |
| MS14 | Eisen, RJ, Eisen, L, Beard, CB. (2016). County-Scale Distribution of Ixodes scapularis and Ixodes pacificus (Acari: Ixodidae) in the Continental United States. J. Med. Entomol. 53(2): 349-386. doi:10.1093/jme/tjv237 |
| MS15 | Chen, D, Wong, H, Belanger, P, Moore, K, Peterson, M, Cunningham, J. (2015). Analyzing the correlation between deer habitat and the component of the risk for Lyme disease in eastern Ontario, Canada: A GIS-based approach. ISPRS. Int. J. Geo-Inf. 4: 105-123. doi:10.3390/ijgi4010105 |
| MS16 | Ashley, ST, Meentemeyer, V. (2004). Climatic analysis of Lyme diseases in the United States. Clim. Res. 27: 177-187. |
| MS17 | Ozdenerol, E, Bingham-Byrne, RM, Seboly, JD. (2021). The effects of lifestyle on the risk of Lyme disease in the United States: Evaluation of market segmentation systems in prevention and control strategies. Int. J. Environ. Res. Public Health. 18: 12883. doi: 10.3390/ijerph182412883 |
| MS18 | Bouchard, C, Aenishaenslin, C, Rees, EE, Koffi, JK, Pelcat, Y, Ripoche, M, Milord, F, Lindsay, LR, Ogden, NH, Leighton, PA. (2018). Integrated social-behavioral and ecological risk maps to prioritize local public health responses to Lyme disease. Env. Health. Perspect. 126(4): 047008. doi: 10.1289/EHP1943 |
| MS19 | Rosen, ME, Hamer, SA, Gerhardt, RR, Jones, CJ, Muller, LI, Scott, MC, Hickling, GJ. (2012). Borrelia burgdorferi not detected in widespread Ixodes scapularis (Acari: Ixodidae) collected from white-tailed deer in Tennessee. J. Med. Entomol. 49(6): 1473-1480. doi: 10.1603/ME11255 |
| MS20 | Ripoche, M, Gasmi, S, Adam-Poupart, A, Koffi, JK, Lindsay, LR, Ludwig, A, Milord, F, Ogden, NH, Thivierge, K, Leighton, PA. (2018). Passive tick surveillance provides an accurate early signla of emerging Lyme disease risk and human cases in southern Canada. J. Med. Entomol. 55(4): 1016-1026. doi: 10.1093/jme/tjy030 |
| MS21 | Aenishaenslin, C, Charland, K, Bowser, N, Perez-Trejo, E, Baron, G, Milord, F, Bouchard, C. (2022). Behavioural risk factors associated with reported tick exposure in a Lyme disease high incidence region in Canada. BMC. Pub. Health. 22: 807. doi: 10.1186/s12889-022-13222-9 |
| MS22 | Ripoche, Lindsay, LR, Ludwig, A, Ogden, NH, Thivierge, K, Leighton, PA. (2018). Multi-scale clustering of Lyme disease risk at the expanding leading edge of the range of Ixodes scapularis in Canada. Int. J. Environ. Res. Public Health. 15: 603. doi:10.3390/ijerph15040603 |
| MS23 | Cheng, A, Chen, D, Woodstock, K, Ogden, NH, Wu, X, Wu, J. (2017). Analyzing the Potential Risk of Climate Change on Lyme Disease in Eastern Ontario, Canada Using Time Series Remotely Sensed Temperature Data and Tick Population Modelling. Remote. Sens. 9:609. doi: 10.3390/rs9060609 |
| MS24 | Neupane, N, Goldbloom-Helzner, A, Arab, A. (2021). Spatio-temporal modeling for confirmed cases of Lyme disease in Virginia. Ticks. Tick. Borne. Dis. 12(6): 101822. doi: 10.1016/j.ttbdis.2021.101822. |
| MS25 | Self, SCWm Liu, Y, Nordone, SK, Yabsley, MJ, Walden, HS, Lund, RB, Bowman, DD, Carpenter, C, McMahan, CS, Gettings, JR. (2019). Canine vector-borne disease: mapping and the accuracy of forecasting using big data from the veterinary community. Animal Health Research Reviews. 20: 47-60. doi: 10.1017/51466252319000045 |
| MS26 | Glavanakov, S, White, DJ, Caraco, T, Lapenis, A, Robinson, GR, Szymanski, BK, Maniatty, WA. (2001). Lyme disease in New York State: Spatial Pattern at a regional scale. AM. J. Trop. Med. Hyg. 65(5): 538-545. |
| MS27 | Gonzalez-Salazar, C, Stephens, CR, Meneses, AK. (2021). Assessment of the potential establishment of Lyme endemic cyclies in Mexico. J. Vect. Eco. 46(2): 207-220. doi: 10.52707/1081-1710-46.2.207 |
| MS28 | Kutera, M, Berke, O, Sobkowich, K. (2022). Spatial epidemiological analysis of Lyme disease in southern Ontario utilizing Google Trends searches. EHR. 64(4): 105-110. doi: 10.5864/d2021-025 |
| MS29 | Cull, B. (2022). Monitoring Trends in Distribution and Seasonality of Medically Important Ticks in North America Using Online Crowdsourced records from iNaturalist. Insects.13: 404. doi: 10.3390/insects13050404 |
| MS30 | Diuk-Wasser, MA, Gatewood, AG, Cortinas, MR, Yaremych-Hamer, S, Tsao, J, Kitron, U, Hickling, G, Brownstein, JS, Walker, E, Piesman, J, Fish, D. (2006). Spatiotemporal patterns of host-seeking Ixodes scapularis nymphs (Acari: Ixodidae) in the United States. J. Med. Entomol. 43(2): 166-176. |
| MS31 | Tran, T, Prusinski, MA, White, JL, Falco, RC, Kokas, J, Vinci, V, Gall, WK, Tober, KJ, Haight, J, Oliver, J, Sporn, LA, Meehan, L, Banker, E, Backenson, PB, Jensen, ST, Brisson, D. (2022). Predicting spatio-temporal population patterns of Borrelia burgdorferi, the Lyme disease pathogen. J. App. Ecol. 59: 2779-2789. doi: 10.1111/1365-2664.14274 |
| MS32 | Guillot, C, Bouchard, C, Buhler, K, Dumas, A, Milord, F, Ripoche, M, Pelletier, Leighton, P. (2022). Sentinel surveillance contributes to tracking Lyme disease spatiotemporal risk trends in Southern Quebec, Canada. Pathogens. 11:531. doi: 10.3390/pathogens11050531 |
| MS33 | Porter, WT, Motyka, PJ, Wachara, J, Barrand, ZA, Hmood, Z, McLaughlin, M, Pemberton, K, Nieto, NC. (2019). Citizen science informs human-tick exposure in the Northeastern United States. Int. J. Health. Geogr. 18:9. |
| MS34 | VanAcker, MC, Little, EAH, Molaei, G, Bajwa, WI, Diuk-Wasser, MA. (2019). Enhancement of risk for Lyme disease by landscape connectivity, New York, New York, USA. Emerg. Infect. Dis. 25(6): 1136-1143. doi: 10.3201/eid2506.181741 |
| MS35 | Diuk-Wasser, MA, VanAcker, MC, Fernandez, MP. (2021). Impact of land use changes and habitat fragmentation on the eco-epidemiology of tick-borne diseases. J. Med. Entomol. 58(4): 1546-1564. doi: 10.1093/jme/tjaa209 |
| MS36 | Larsen, AE, MacDonald, AJ, Plantinga, AJ. (2014). Lyme disease risk influences human settlement in the wildland-urban interface: evidence from a longitudinal analysis of counties in the Northeastern United States. Am. J. Trop. Med. Hyg. 91(4): 747-755. doi:10.4269/ajtmh.14-0181 |
| MS37 | Slatculescu, AM, Duguay, C, Ogden, NH, Sander, B, Desjardins, M, Cameron, DW, Kulkarni, MA. (2022). Spatiotemporal trends and socioecological factors associated with Lyme disease in eastern Ontario, Canada from 2010-2017. BMC Public Health. 22:736. doi: 10.1186/s12889-022-13167-z |

**Supplementary Table 2.** List of individual variables recorded from the literature review data collected from the 61 research articles focused on. Variables are separated by their variable group and organized from most frequent to least within each group. Similar variables were grouped together.

| Variable Group | Variable | Frequency |
| --- | --- | --- |
| Ecological | Tick occurrence (presence/absence) | 21 |
|  | Human Lyme disease case data | 18 |
|  | Tick abundance/density | 17 |
|  | *Borrelia* prevalence in ticks | 12 |
|  | Non-human host density/distribution/*Borrelia* prevalence | 6 |
|  | Tick distribution (from older source) | 1 |
|  | Local dispersal and long-distance dispersal of ticks | 1 |
|  | Simulated tick populations | 1 |
|  | Tick development rate and mortality at each life stage | 1 |
|  | Borrelia genetic variations/variants | 1 |
|  |  |  |
| Environmental | Temperature | 28 |
|  | Land cover | 18 |
|  | Elevation/altitude | 13 |
|  | Precipitation | 13 |
|  | Vegetation index/forest cover | 10 |
|  | Vapour pressure/humidity | 7 |
|  | Soil properties | 4 |
|  | Aspect/slope | 2 |
|  | Eco divisions | 2 |
|  | Habitat connectivity | 2 |
|  | Wind circulation | 1 |
|  | Water cover | 2 |
|  |  |  |
| Anthropic | Human pop density/size | 8 |
|  | Behaviour survey | 3 |
|  | Knowledge score | 1 |
|  | Household income | 1 |
|  | Patient age | 1 |
|  | Patient sex | 1 |
|  | "Lifestyle" score | 1 |
|  | Risk perception score | 1 |

**Supplementary Table 3.** The cumulative number of anthropic, environmental, and ecological variables used across years (2000-2022) in the reviewed studies; no studies found for 2000 and 2007. The total number of variables for each variable group is the sum of those variables used across studies (n = 61). Numbers in brackets indicate the number of studies for that specific year.

| Year | Total Anthropic Variables Used | Total Environmental Variables Used | Total Ecological Variables Used |
| --- | --- | --- | --- |
| 2001 (1) | 1 | 0 | 1 |
| 2002 (2) | 0 | 2 | 2 |
| 2003 (1) | 0 | 2 | 1 |
| 2004 (1) | 0 | 3 | 1 |
| 2005 (1) | 0 | 3 | 1 |
| 2006 (2) | 0 | 1 | 2 |
| 2008 (1) | 0 | 2 | 1 |
| 2009 (1) | 0 | 4 | 2 |
| 2010 (1) | 0 | 4 | 1 |
| 2011 (1) | 2 | 1 | 0 |
| 2012 (3) | 0 | 8 | 5 |
| 2013 (1) | 0 | 1 | 1 |
| 2014 (4) | 1 | 7 | 5 |
| 2015 (1) | 0 | 3 | 2 |
| 2016 (3) | 0 | 3 | 5 |
| 2017 (4) | 0 | 8 | 3 |
| 2018 (8) | 5 | 13 | 10 |
| 2019 (4) | 3 | 5 | 8 |
| 2020 (4) | 1 | 10 | 8 |
| 2021 (8) | 4 | 13 | 11 |
| 2022 (9) | 3 | 9 | 12 |

**Supplementary Table 4.** Literature review data collected from 61 research articles where their results included risk maps. Here, the variables used in those maps were categorized as either ecological (Ecol), environmental (Enviro), or anthropic (Anthro) and tallied. Distribution = map distribution focused on host (humans) or vector (ticks). Life Stage = tick life stage focused on to produce the risk models. Tick surveillance = methodology used to collect tick data (if applicable).  

| Paper ID | Year | Map Type | Distribution | Tick Surveillance | Scale | Life Stage | Country |
| --- | --- | --- | --- | --- | --- | --- | --- |
| MP1 | 2022 | Predictive | Vector | Active | local | Nymph | USA |
| MP2 | 2008 | Predictive | Vector | Passive | national | Nymph | Canada |
| MP3 | 2012 | Predictive | Vector | Passive | national | Immature | Canada |
| MP4 | 2006 | Predictive | Vector | N/A | national | N/A | Canada |
| MP5 | 2002 | Predictive | Vector | Active | local | All stages | USA |
| MP6 | 2020 | Predictive | Vector and Host | Active and Passive | local | All stages | Canada |
| MP7 | 2017 | Predictive | Vector | Active | regional | All stages | Canada |
| MP8 | 2022 | Predictive | Vector | N/A | national | All stages | USA, Canada, Mexico |
| MP9 | 2018 | Predictive | Vector | Passive | local | Adult | Canada |
| MP10 | 2012 | Predictive | Vector and Host | Active | regional | Nymph | USA |
| MP11 | 2018 | Predictive | Host | N/A | local | N/A | USA |
| MP12 | 2003 | Predictive | Vector | Active | regional | All stages | USA |
| MP13 | 2013 | Predictive | Vector | Active | national | All stages | Canada |
| MP14 | 2018 | Predictive | Vector | Passive | local | All stages | Canada |
| MP15 | 2020 | Predictive | Host | Passive | local | All stages | USA |
| MP16 | 2005 | Predictive | Vector | Active and Passive | national | All stages | USA and Canada |
| MP17 | 2022 | Predictive | Vector | Passive | local | All stages | Canada |
| MP18 | 2019 | Predictive | Vector and Host | Passive | local | Nymph | USA |
| MP19 | 2010 | Predictive | Vector | Active | regional | Nymph | USA |
| MP20 | 2018 | Predictive | Vector | Active | local | Nymph | USA |
| MP21 | 2017 | Predictive | Vector | N/A | regional | N/A | Canada |
| MP22 | 2020 | Predictive | Host | N/A | local | All stages | USA |
| MP23 | 2014 | Predictive | Vector | N/A | regional | N/A | USA and Canada |
| MP24 | 2014 | Predictive | Vector | Active and Passive | regional | N/A | USA |
| MS1 | 2016 | Surveillance | Vector | Active | local | All stages | USA |
| MS2 | 2002 | Surveillance | Host | N/A | local | N/A | USA |
| MS3 | 2021 | Surveillance | Host | N/A | local | N/A | Canada |
| MS4 | 2016 | Surveillance | Vector and Host | Active | local | All stages | Canada |
| MS5 | 2018 | Surveillance | Vector and Host | Passive | local | Adult | USA |
| MS6 | 2021 | Surveillance | Vector | Active | regional | All stages | Canada |
| MS7 | 2022 | Surveillance | Vector | Active and Passive | local | All stages | USA |
| MS8 | 2011 | Surveillance | Host | N/A | local | N/A | Canada |
| MS9 | 2017 | Surveillance | Host | N/A | local | N/A | USA |
| MS10 | 2014 | Surveillance | Vector and Host | Passive | local | All stages | USA |
| MS11 | 2009 | Surveillance | Vector | Active and Passive | local | Nymph | USA |
| MS12 | 2019 | Surveillance | Vector and Host | Passive | local | All stages | Canada |
| MS13 | 2020 | Surveillance | Vector | Active and Passive | local | All stages | Canada |
| MS14 | 2016 | Surveillance | Vector | Active and Passive | local | All stages | USA |
| MS15 | 2015 | Surveillance | Vector | Passive | local | All stages | Canada |
| MS16 | 2004 | Surveillance | Host | N/A | local | N/A | USA |
| MS17 | 2021 | Surveillance | Host | Active | regional | All stages | USA |
| MS18 | 2018 | Surveillance | Host | Active | local | All stages | Canada |
| MS19 | 2012 | Surveillance | Vector and Host | Passive | local | Adult | USA |
| MS20 | 2018 | Surveillance | Vector and Host | Passive | local | All stages | Canada |
| MS21 | 2022 | Surveillance | Vector | N/A | local | N/A | Canada |
| MS22 | 2018 | Surveillance | Vector | Active | local | Nymph | Canada |
| MS23 | 2017 | Surveillance | Vector and Host | Passive | local | All stages | USA |
| MS24 | 2021 | Surveillance | Host | N/A | local | N/A | Canada |
| MS25 | 2019 | Surveillance | Host | Passive | regional | All stages | USA |
| MS26 | 2001 | Surveillance | Host | N/A | regional | N/A | USA, Mexcio |
| MS27 | 2021 | Surveillance | Vector and Host | Active and Passive | national | All stages | Canada |
| MS28 | 2022 | Surveillance | Vector | N/A | regional | All stages | USA and Canada |
| MS29 | 2022 | Surveillance | Vector and Host | Active and Passive | national | All stages | USA |
| MS30 | 2006 | Surveillance | Vector | Active | regional | Nymph | USA |
| MS31 | 2021 | Surveillance | Vector | Active | regional | Nymph | Canada |
| MS32 | 2022 | Surveillance | Vector and Host | Active | local | Nymph | USA |
| MS33 | 2019 | Surveillance | Vector | Passive | regional | All stages | USA |
| MS34 | 2021 | Surveillance | Vector | Active | local | All stages | USA |
| MS35 | 2021 | Surveillance | Host | N/A | regional | N/A | USA |
| MS36 | 2014 | Surveillance | Host | N/A | local | N/A | Canada |
| MS37 | 2022 | Surveillance | Vector and Host | Passive | local | All stages | Canada |

**Supplementary Table 5.** Non-metric multidimensional scaling (NMDS) centroid positions of map elements classifying studies based on the frequency of ecological, environmental, and anthropic variables incorporated in each risk map model (n = 61). Variables include Year of publication, Map Type, Map Distribution, Tick Life Stage, Country map is based in, and Map Scale. Number of permutations = 999. USA = United States of America. N/A = maps without this data.

| **Variables** | **NMDS1** | **NMDS2** |
| --- | --- | --- |
| Year of Publication | 0.58442 | 0.81145 |
| Map Type (Predictive) | -0.3981 | -0.0334 |
| Map Type (Surveillance) | 0.2582 | 0.0217 |
| Distribution (Host only) | 0.1486 | 0.2597 |
| Distribution (Vector only) | -0.1725 | -0.0828 |
| Distribution (Host & Vector) | 0.2351 | -0.0890 |
| Tick Life Stage (Adult) | 0.2798 | -0.1363 |
| Tick Life Stage (All Stages) | -0.0107 | 0.0051 |
| Tick Life Stage (Immature) | -0.5609 | -0.0008 |
| Tick Life Stage (N/A) | 0.01216 | 0.0417 |
| Tick Surveillance (Passive) | 0.0340 | -0.0158 |
| Tick Surveillance (Active) | -0.1379 | -0.0513 |
| Tick Surveillance (Active & Passive) | -0.0019 | -0.1004 |
| Tick Surveillance (N/A) | 0.1129 | 0.1232 |
| Scale (National) | -0.2154 | -0.1908 |
| Scale (Regional) | -0.0572 | 0.0635 |
| Scale (Local) | 0.0713 | 0.0138 |
| Country (Canada) | 0.0063 | 0.0602 |
| Country (USA) | -0.0045 | -0.0710 |
| Country (USA & Canada) | -0.1408 | 0.1514 |
| Country (USA and Mexico) | 0.7945 | -0.1986 |
| Country (USA, Canada, and Mexico) | -0.4008 | 0.3111 |
